# Temperature/pH-Sensitive Double Cross-Linked Hydrogels as Platform for Controlled Delivery of Metoclopramide

**DOI:** 10.3390/gels8120824

**Published:** 2022-12-13

**Authors:** Bogdan-Paul Coșman, Sanda-Maria Bucătariu, Marieta Constantin, Gheorghe Fundueanu

**Affiliations:** “Petru Poni” Institute of Macromolecular Chemistry, Gr. Ghica Voda Alley 41A, 700487 Iasi, Romania

**Keywords:** smart hydrogels, double cross-linked, drug delivery systems

## Abstract

Novel double cross-linked (DC) hydrogels with pH-/temperature-sensitive properties were designed and developed. Therefore, linear pH-sensitive poly(methyl vinyl ether-alt-maleic acid) (P(VME/MA)) macromolecules were absorbed within a thermosensitive poly(*N*-isopropylacrylamide-co-hydroxyethylacrylamide)-hydrogel (PNH) and, subsequently, cross-linked together through a solvent-free thermal method. As a novelty, double cross-linked hydrogels were obtained from previously purified polymers in the absence of any solvent or cross-linking agent, which are generally harmful for the body. The new DC structures were characterized by FT–IR spectroscopy, SEM, swelling kinetic measurements, and mechanical tests. The resulting scaffolds exhibited interconnected pores and a flexible pattern, compared to the brittle structure of conventional PNH. The swelling kinetics of DC hydrogels were deeply affected by temperature (25 and 37 °C) and pH (7.4 and 1.2). Furthermore, the hydrogels absorbed a great amount of water in a basic environment and displayed improved mechanical properties. Metoclopramide (Met) was loaded within DC hydrogels as a model drug to investigate the ability of the support to control the drug release rate. The results obtained recommended them as convenient platforms for the oral administration of drugs, with the release of the largest part of the active principle occurring in the colon.

## 1. Introduction

Hydrogels are three-dimensional (3D) networks made of natural, synthetic or semi-synthetic polymers, physically or chemically cross-linked, which can absorb a large amount of water and biological fluids [1,2]. This specific capacity is due to the existence of hydrophilic groups in the polymer network, such as amino, hydroxyl, carboxyl, sulfonate, etc. [3]. These functional groups are responsible for the formation of non-covalent interactions with various natural tissues [3,4]. The structure and physicochemical properties of hydrogels can be developed and controlled by choosing the appropriate biomaterials, cross-linking methods, and fabrication strategies [5]. As mentioned above, these 3D structures can be obtained by physical or chemical cross-linking. The physically cross-linked hydrogels are obtained through secondary hydrogen linking amongst polar groups in the polymer network, while the chemically cross-linked hydrogels are formed by molecular bonds of distinct functional groups in the polymer network, mediated by specific cross-linking agents, such as glutaraldehyde, *N*,*N*′-methylenebisacrylamide, etc. [6].

Hydrogels based on intelligent polymers exhibit sensitivity to small changes in the external medium and display responses in varying their form or volume when exposed to particular conditions. The polymeric networks are responsive to physical, chemical, and biological stimuli. Among them, the most common stimuli are light [7], temperature [8,9], electric and magnetic fields, ultrasound, pH, redox, ionic strength, CO_2_, glucose, enzymes, antigens, glutathione, and DNA [10]. Hydrogels can be safely applied as devices in tissue engineering, due to their hydrophilicity, viscoelasticity, biodegradability and similarity to the extracellular matrix [11,12]. Moreover, this class of materials is frequently used as a support for drug delivery, because it presents high biocompatibility [13], spatiotemporal control of drug release, protects the bioactive compound from harsh conditions in the body [14], display physicochemical adaptability [15], etc. Amongst stimuli responsive hydrogels, temperature and pH sensitive ones are the most used for biomedical applications, because they exploit change in the temperature and pH of the human body [10].

Thermo-responsive hydrogels exhibit reversible swelling and shrinking behavior, depending on temperature changes [3]. Poly(*N*-isopropylacrylamide) (PNIPAAm) is the first and most studied thermosensitive synthetic polymer [8,9,16]. PNIPAAm presents a lower critical solution temperature (LCST) in aqueous solutions at approximately 32 °C, a value that can be adjusted by copolymerization with hydrophilic or hydrophobic monomers [17,18,19]. The introduction of hydrophilic monomers generally increases the LCST, while the incorporation of hydrophobic units/groups has the opposite effect [20,21]. However, PNIPAAm-based hydrogels are limited in their biomedical applications because they exhibit low biocompatibility and biodegradability [20,22]. Overcoming these impediments can be done by copolymerization, grafting PNIPAAm on different natural or biocompatible polymers such as the following: hyaluronic acid [23], chitosan [24], Gantrez [25], etc. In order to design optimal drug carriers, natural hydrogels can be combined with synthetic ones to obtain supports with synergic properties [14]. The pH-sensitive hydrogels exhibit a special affinity for biomedical applications, especially as drug delivery platforms [1]. These 3D structures have the ability to respond to alterations of the pH along the gastrointestinal tract and different cellular compartments, through structural and functional changes, such as modification of surface activity, conformation, solubility and configuration [20]. The pH-sensitive hydrogels are obtained from polymers with ionizable acidic or basic groups in the main chain, or as pendant groups that are able to accept or donate protons [1,21,26]. The properties of these hydrogels are controlled by various factors, such as the nature of functional groups, ionic strength, pK_a_ or pK_b_, wettability, macromolecule concentration. For example, chitosan and poly(ethylene imine)–based hydrogels [27], swell at low pH as a consequence of protonation of the amino/imine groups. In fact, the protonated positively charged groups cause repulsion between the polymeric chains and extension of the 3D network. On the other hand, anionic hydrogels, made of carboxymethyl chitosan [28] or carboxymethyl pullulan [29], swell at basic pH, due to ionization of the acidic groups. The negatively charged ionized groups cause repulsion between macromolecules and, therefore, swelling of the hydrogel. This characteristic of anionic hydrogels is exploited to deliver drugs in intestines [30].

The composition and inner structure of the hydrogels are essential characteristics when they are designed for tissue engineering applications [2,3,16,31]. The scaffold should supply temporary mechanical and non-toxic support for the cells [31]. On this matter, the hydrogels should provide a tunable porosity and a soft three-dimensional network that is similar to the connective tissue [32]. In order to enhance the biodegradability of PNIPAAm hydrogels, poly(ethylene glycol) (PEG) [5] and poly(ε-caprolactone) (PCL) [19] are the most applicable, whereas biocompatibility is mostly accomplished with biopolymers [12,19]. Most PNIPAAm-based hydrogels are weak and brittle with low strength (low value of Young modulus (5–10 kPa)) [33]. The design of strong and resilient hydrogels would expand their applications to innovative areas, such as actuators [34], robotics [33], self-folding [35], etc. [9].

Poly(methyl vinyl ether-co-maleic anhydride), (P(VME/MA)) is a biodegradable and biocompatible linear polymer and presents low toxicity [36,37], and is authorized by the FDA. This copolymer, industrially known as Gantrez (G), was employed in designing dental adhesives [25], scaffolds for tissue engineering [38], and in microneedle arrays for drug delivery [39]. Moreover, the copolymer is biocompatible, having a large number of carboxylic groups. By combining P(MVE/MA) with other macromolecules containing alcohol groups, an esterification reaction occurs, resulting in biomaterials with improved properties [37].

The cross-linked network of hydrogels used as drug transport systems can protect the drugs against enzymes or harsh environments, like low pH in the stomach [1]. Adjustment of their cross-linking degree leads to better optimization of drug loading and release [1,40] as well as improving the mechanical properties of the hydrogels [40]. This can be achieved by using more than one cross-linker, which generally reduces the degree of swelling of the reagents [1]. However, if one of the cross-linkers contains hydrophilic groups, the obtained hydrogels show higher degrees of swelling, compared with hydrogels cross-linked with hydrophobic reagents.

Che et al. [41] obtained an interesting hydrogel, based on polyacrylamide (PAM) and poly(2-methyl-2-oxazoline), through a double cross-linking approach. In order to obtain on-demand tailored mechanical properties, an inclusion complex, based on adamantane and β-CD, was used as a second cross-linker [41]. Chen et al. [42] obtained a hydrogel based on quaternized chitosan and PAM using polydopamine as a novel connecting bridge. The authors showed that both covalent and physical cross-linking methods improved the mechanical stability and self-healing capacity of the hydrogels [42]. Li et al. [40] obtained a hydrogel based on poly(2-acrylamido-2-methylpropane sulfonic acid-co-*N*,*N*-dimethylacrylamide) double cross-linked with Laponite and graphene oxide. The resulting hydrogel exhibited intrinsic ultra-elasticity and excellent healing performance, either under heating or infrared light treatment [40].

In the present study, novel pH/thermosensitive double cross-linked (DC) hydrogels were prepared by the inclusion of pH-sensitive P(VME/MA) linear copolymer within the cross-linked network of thermosensitive poly(*N*-isopropylacrylamide-co-hydroxyethylacrylamide) (PNH). Subsequently, P(VME/MA) was fixed by additional cross-linking through a solvent-free thermal method. The novel pH-/thermo-sensitive hydrogels were characterized by SEM, FTIR and in terms of swelling/deswelling kinetics. The effect of the polymer concentration P(MVE/MA) on the mechanical properties of DC hydrogels was investigated. To test the capacity of these hydrogels to control the delivery of drugs, in vitro release studies of metoclopramide (Met) were carried out in buffer solutions simulating physiological conditions.

## 2. Results and Discussion

### 2.1. Synthesis and Characterization of Conventional Poly(NIPAAm-co-HEAAm) (PNH) and Double Cross-Linked Hydrogels

**Figure 1:**
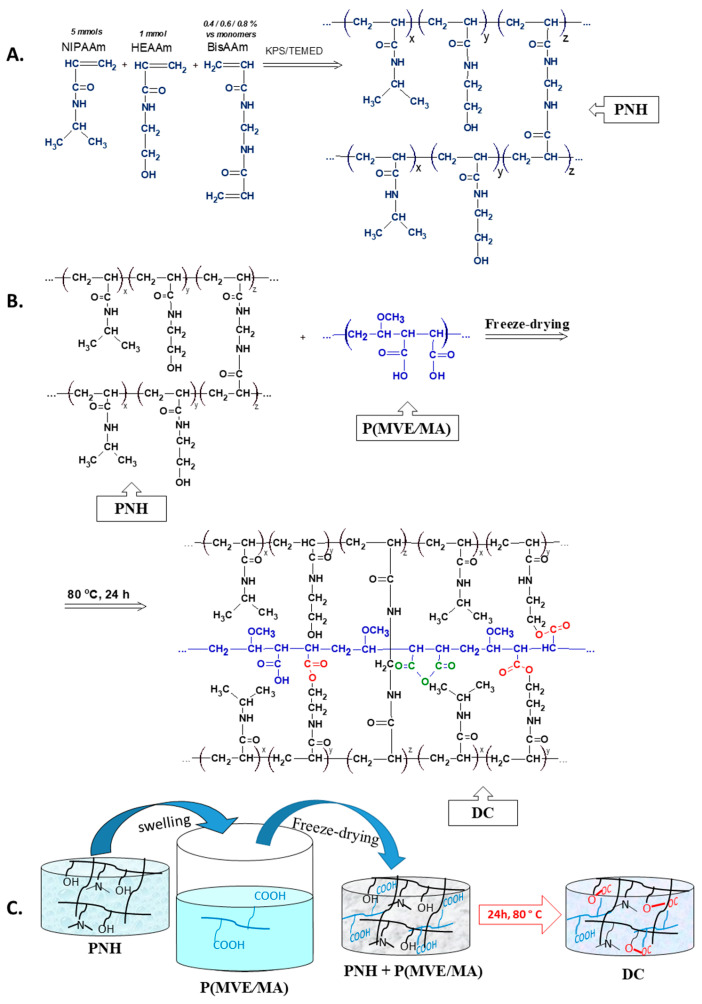
Chemical reactions involved in the synthesis of conventional (**A**) and double cross-linked (**B**) hydrogels. Illustrative depiction of the hydrogel synthesis (**C**).

Conventional poly(NIPAAm-co-HEAAm) hydrogels were prepared by copolymerization of 5 mmol of NIPAAm and 1 mmol of HEAAm in the presence of BisAAm as a cross-linker (Figure 1A). This molar ratio was already established by Fundueanu et al. [43], in order to obtain copolymers with a lower critical solution temperature (LCST) value, close to that of the human body [44]. HEAAm was selected as the comonomer since its molecule contains a hydrophilic, secondary alcohol group that can be involved in covalent cross-linking. The synthesis parameters of conventional and double cross-linked hydrogels are presented in Table 1. Thus, conventional hydrogels were immersed in P(MVE/MA) solutions of different concentrations (2 and 5%, *w*/*v*) until equilibrium was reached, and then the swollen hydrogels were lyophilized. The obtained semi-interpenetrated polymeric samples had a cylindrical shape, being formed first by a cross-linked network based on poly(NIPAAm-co-HEAAm) including free linear polymers(P(MVE/MA)). To link the free polymer to the first network, the lyophilized samples were placed in an oven at 80 °C for 24 h. At this temperature, condensation reactions occurred between the hydroxyl groups of PNH and the carboxyl groups of P(MVE/MA), resulting in ester bonds (Figure 1B). Moreover, at this temperature, the water could be removed between the adjacent carboxyl groups of P(MVE/MA), resulting in anhydrides [44]. The cross-linking method occurring in the dried state offered several advantages over conventional reactions in solutions. One of the most important advantages was the fact that no solvent was necessary, and, thus, it was an eco-friendly method. The synthesis steps of DC hydrogels are illustrated in Figure 1. Both PNH and DC hydrogels were successfully prepared with a yield of 85–90%. The samples were noted in the form DCx-Py, where “x” represents the BisAAm percentage (BisAAm vs. monomers, % mmol) and “y” indicates the concentration of P(MVE/MA) (P) (%, *w*/*w*) in the initial mixture. The composition of the initial reaction mixture and of the DC hydrogels is given in Table 1.

**Figure 2:**
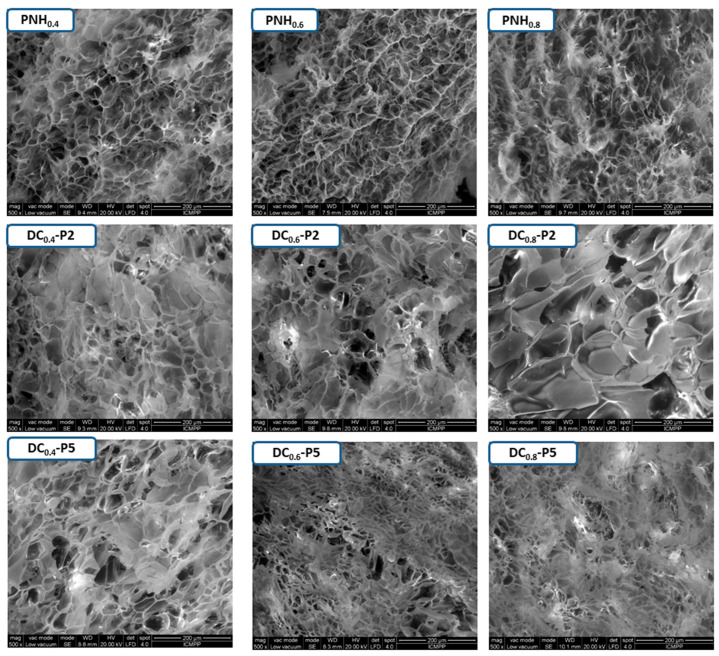
SEM micrographs of conventional and double cross-linked hydrogels. Magnification ×500.

The synthesized DC–P hydrogels presented different balances between hydrophilic (–CONH– and –(COOH)_2_ pendant groups) and hydrophobic (–CH(CH_3_)_2_ groups) interactions. In fact, the hydrophilic and hydrophobic groups of the P(MVE/MA) interacted with the preformed PNH network during the cross-linking reaction. Thereby, the DC hydrogels presented different morphologies, thermal, mechanical and swelling properties. As follows, the lyophilized PNH and DC hydrogels were analyzed in terms of morphology by SEM microscopy (Figure 2).

It is worth mentioning that the addition of P(MVE/MA) into conventional PNH hydrogels (Figure 2) notably changed the inner aspect of the pores. Since the DC hydrogels were obtained from the preformed PNH network, it was expected that the size of the pores would remain the same but many would be filled with P(MVE/MA), the degree of filling being dependent on the concentration of P(MVE/MA). Along with structure information, these images showed a pretty homogeneous interpenetrating structure. These results may be attributed to a good diffusion of P(MVE/MA) in the swollen PNH at all the compositions and at both concentrations of linear polymer. In addition, intermolecular H-bonding interactions between P(MVE/MA) and PNH occurred, stabilizing the network.

The DC hydrogels obtained from conventional ones cross-linked with 0.6% BisAAm proved to be the most convenient to be studied further. They had approximately the same amount of linked P(MVE/MA) as those cross-linked with 0.4% BisAAm but higher than those with 0.8% BisAAm (Table 1). From a stability point of view, they were preferable to those cross-linked with 0.4% BisAAm.

The porosity of DC hydrogels decreased with the increase of the P(MVE/MA) amount, as can be seen in Table 2. In fact, the higher the percentage of P(MVE/MA) in the hydrogel the more pores were filled and the less void spaces were available.

The degree of the second cross-linking was indirectly determined by the conductometric titration of carboxylic groups, calculated as the difference between the theoretical and effective exchange capacities. The obtained data are reported in Table 2.

**Figure 3:**
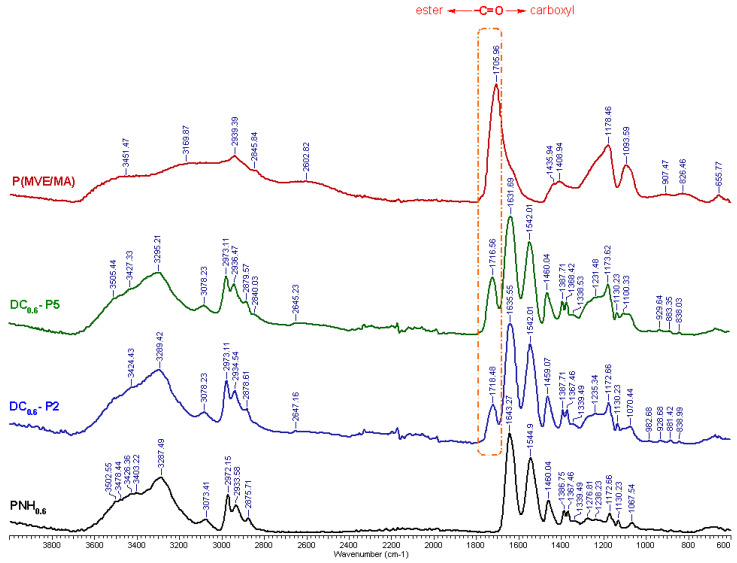
FT-IR spectra of the conventional (PNH_0.6_) and double cross-linked hydrogels (DC_0.6_–P2 and DC_0.6_–P5). For comparison the spectrum of P(MVE/MA) is depicted.

The comparison of the FTIR spectra of the conventional PNH and P(MVE/MA) with those of double cross-linked hydrogels (Figure 3) confirmed the presence of P(MVE/MA) and the appearance of ester bonds in the final product. The spectrum of the PNH_0.6_ hydrogel showed a band from 3600 to 3050 cm^−1^, related to the stretching vibration of hydroxyl groups (OH) of HEAAm and amide groups (–NH–CO–) of NIPAAm. In P(MVE/MA), the bands between 2940 and 2845 cm^−1^ were assigned to C–H stretching vibrations of CH, CH_2_, and CH_3_ groups, followed by an –OH specific broadband [25]. The band of the carbonyl groups of P(MVE/MA) shifted from 1705 cm^−1^ to a higher wavenumber (1716 and 1718 cm^−1^) in DC hydrogels [36]. In fact, the shift and broadening of the carbonyl group specific band proved the formation of the ester bonds, as also reported in the literature [36,39]. The formation of these bonds could not be evidenced in the FTIR spectra due to their low concentration [45]. However, the formation of at least 1–2 ester bonds inside the hydrogel was sufficient to form a stable cross-linking network [45]. Moreover, the peak at 1643 cm^−1^ was attributed to the carbonyl group of the amide in PNH hydrogel and this peak shifted at 1635 cm^−1^ in the double cross-linked network.

### 2.2. Thermo-Sensitive Properties

#### 2.2.1. Phase Transition Characterization

It is well established that the most important property of a smart hydrogel is the amplitude of the volume change under the action of environmental stimuli, which mainly depends on the degree of network cross-linking and the density of functional groups [25].

**Figure 4:**
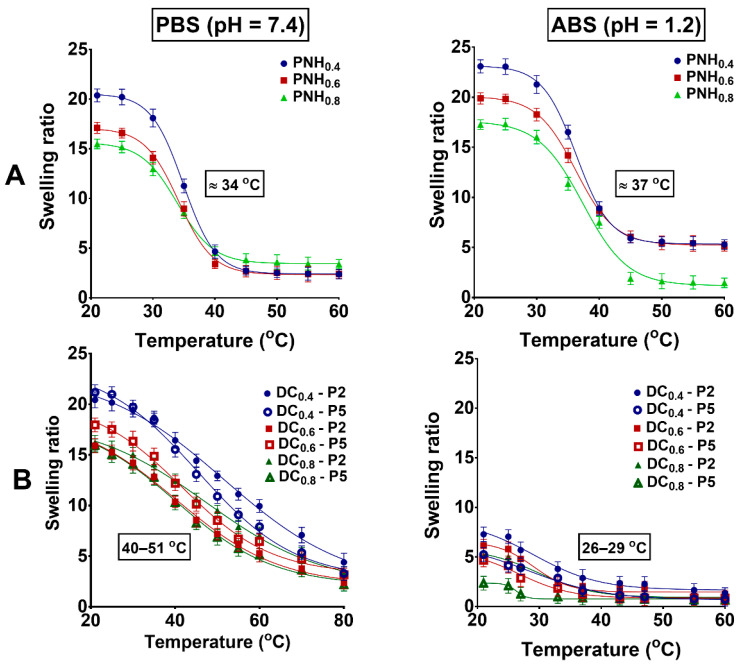
Swelling ratios of conventional (**A**) and DC (**B**) hydrogels as a function of temperature measured in simulated physiological conditions.

**Figure 5:**
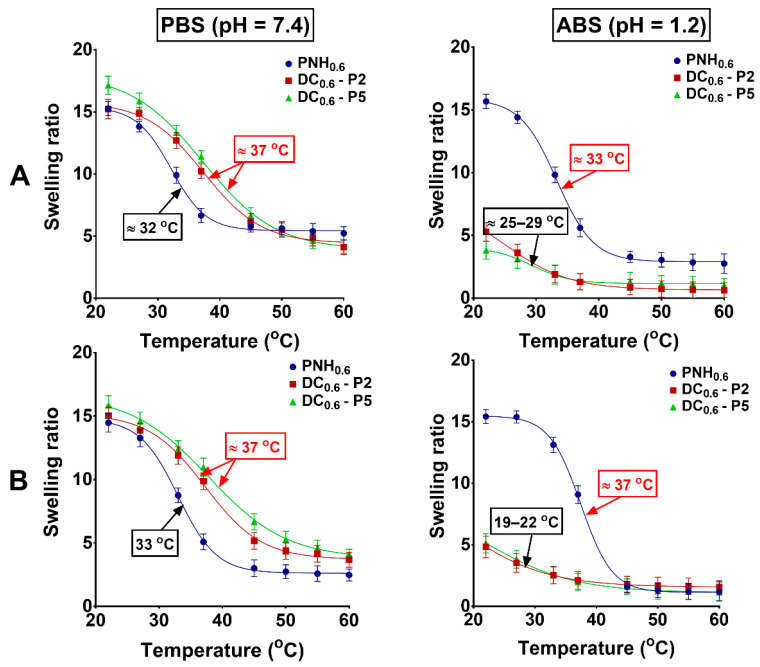
Swelling ratios of conventional and DC hydrogels as a function of temperature in the presence of Met at different concentrations: 1 mg/mL (**A**) and 5 mg/mL (**B**). The measurements were carried out in simulated physiological conditions (PBS at pH = 7.4 and ABS at pH = 1.2).

The swelling ratios (SRs) of both conventional and DC hydrogels are depicted in Figure 4 and Figure 5, respectively. Experiments were carried out in a temperature domain ranging from 25 to 60 °C, in PBS and ABS, in the absence (Figure 4) or presence of Met at different concentrations (Figure 5). The VPTT was determined as the inflection point in the curve swelling factor-temperature by Boltzmann fitting of the experimental data. All the conventional hydrogels showed a relatively sharp volume transition from high to low swelling degrees around the VPTT (34 °C in PBS and 37 °C in ABS). The presence of P(MVE/MA) in DC hydrogels significantly modified the value of VPTT and also influenced the allure of the swelling curves. Due to the hydrophilicity given by the ionization of carboxyl groups in PBS at pH = 7.4, the VPTT values were moved to higher values (40–50 °C) (Figure 4B). The swelling degrees decreased almost linearly and the slopes were less sharp. In acidic buffer (ABS, pH = 1.2) the conventional hydrogels underwent a decrease of the swelling degree with temperature, and the allure of the curves were almost similar to those in PBS (Figure 4A). In fact, since the conventional hydrogels did not possess ionizable groups, the pH should not have influenced the VPTT. However, a slight increase of VPTT was observed (37 °C). Given that the ionic strength was almost the same in the two buffers (PBS and ABS), this increase was due to the easy ionization of the nitrogen in NIPAAm [46]. On the opposite side, the DC hydrogels contained carboxylic groups which, in acidic buffer, were in the protonated state, and more hydrophobic. The swelling degree was low and the difference between the highest and lowest hydrogel volume was much more reduced (Figure 4B).

Surprisingly, in the presence of Met that electrostatically interacted with the new carboxylic groups introduced into the DC network, the hydrogels showed a decrease of the VPTT to a temperature close to that of the human body (Figure 5). Moreover, the slopes of the curves were more abrupt. In fact, as Met is a drug with hydrophobic properties, after electrostatic interactions with the carboxylic groups of maleic acid, the hydrophobicity of the copolymer increased and, therefore, the VPTT decreased. In the acidic medium, the carboxylic groups were in the protonated state and, therefore, the hydrogel practically collapsed. As follows, the influence of the Met was almost negligible at both concentrations of the drug (1 and 5 mg/mL) (Figure 5).

#### 2.2.2. Swelling/Deswelling Kinetics

**Figure 6:**
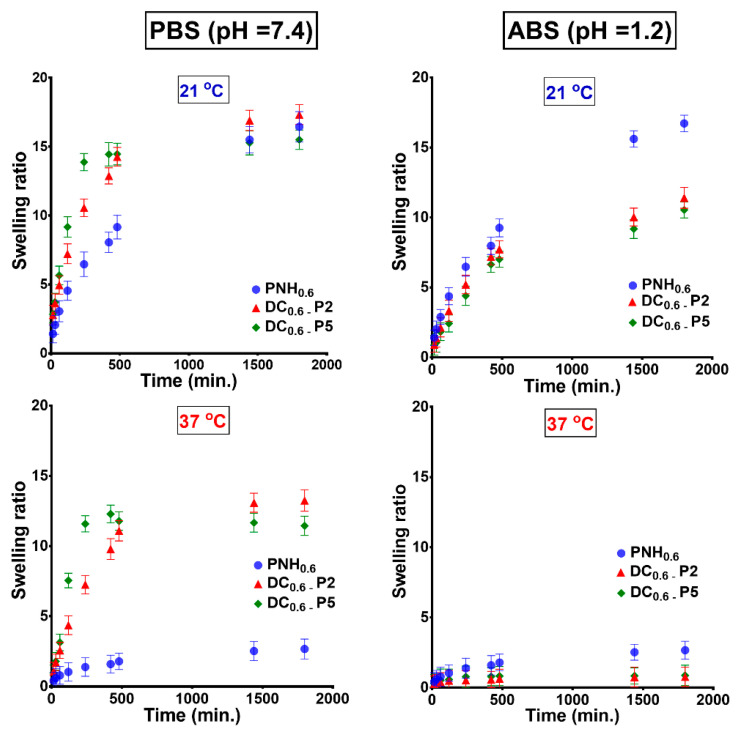
Swelling kinetics of conventional and double cross-linked hydrogels determined in buffer solution at pH 7.4 and pH 1.2 at 21 and 37 °C.

A significant characteristic of the stimuli-sensitive hydrogels used as biomaterials with potential applications in tissue engineering or drug delivery is the rapidity of the swelling/deswelling process when temperature changes below and above the VPTT. Therefore, the swelling kinetics of hydrogels were investigated in simulated physiological conditions (PBS and ABS) in relation to temperatures of 21 °C and 37 °C (Figure 6).

**Figure 7:**
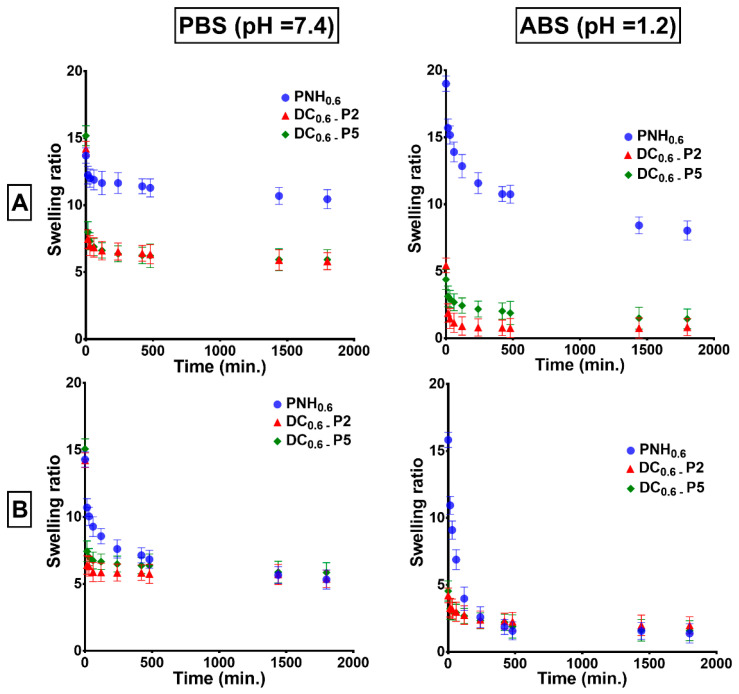
Deswelling kinetics of conventional (PNH_0.6_) and double cross-linked hydrogels measured in buffer solutions in the presence of 1 mg/mL (**A**) and 5 mg/mL (**B**) of Met aqueous solutions.

As shown in Figure 6, at 21 °C the swelling rates of the conventional hydrogels were almost similar in PBS and ABS, both increasing almost linearly. On the contrary, the DC hydrogels swelled more rapid in PBS than in ABS, since the carboxylic groups at pH = 7.4 were in ionized states, and so more hydrophilic. At 37 °C, the swelling rate of conventional hydrogel was low, both in PBS and ABS, because at this temperature the polymeric matrix was in a collapsed state. However, the DC hydrogels swelled faster in PBS because the ionization of the –COOH groups prevented intermolecular hydrogen bonds and favored the expansion of the network. In acidic buffer at 37 °C, the DC hydrogels were almost collapsed and the volume change in time was insignificant. We already demonstrated that, in the presence of the Met, DC hydrogels had a VPTT very close to the temperature of the human body. This behavior is very advantageous since the hydrogels are intended to be used for biomedical applications (drug delivery or tissue engineering). As in the case of the swelling tests, the deswelling studies were performed in simulated physiological conditions in the presence of Met (Figure 7).

As shown in Figure 7, the deswelling rates were much higher than the swelling rates because, during the shrinking process, the water was mechanically expelled from the hydrogel, a process much faster than diffusion within a polymeric network. The deswelling rates, as well as the amplitude of the volume change, increased with increase of Met concentration. Moreover, the deswelling rates were higher in acidic than basic buffer, due to the increase of the network hydrophobicity given by the protonated carboxylic groups. Unexpectedly, even if the conventional hydrogels did not contain ionizable groups, they behaved similarly. In this case, an increase of drug concentration signified a higher ionic strength and, therefore, a more hydrophobic character of the hydrogel due to the desolvation of polymer chains through the “salting out” effect.

**Figure 8:**
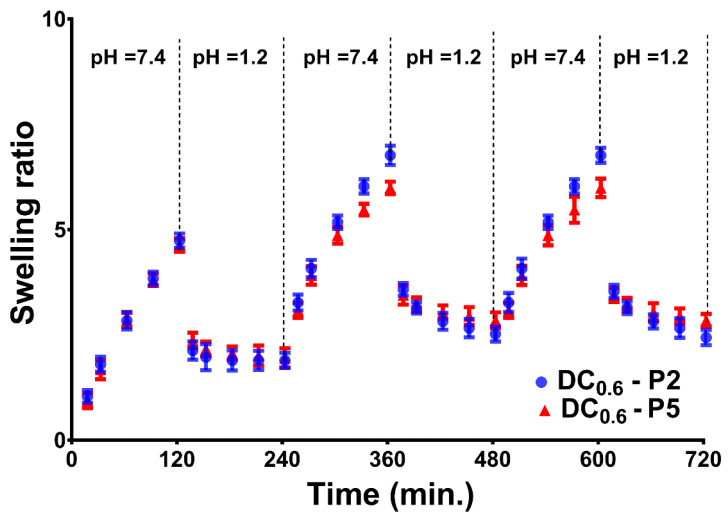
Oscillatory swelling kinetics of DC_0.6_ at 37 °C by alternation of the swelling medium between ABS and PBS.

The swelling/deswelling repeatability of the DC hydrogel between ABS and PBS solutions is depicted in Figure 8. Due to the porous structure, the polymeric 3D structures were capable of absorbing and desorbing the aqueous solution from the medium quickly upon pH change from basic to acidic conditions and the reverse. However, the time for swelling was longer than that for deswelling, because, during protonation of the carboxylic groups, the swollen network collapsed, mechanically expelling a large amount of water, as explained above.

### 2.3. Mechanical Properties

**Figure 9:**
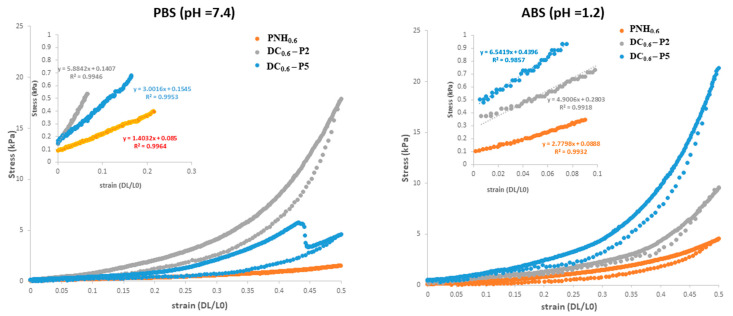
Stress–strain curves for conventional and double cross-linked hydrogels determined in simulated physiological conditions.

The mechanical properties of hydrogels are among the most important characteristics in biomedical applications. Young’s modulus has been highly investigated in this regard [12]. Before the compression tests, the hydrogel samples were swollen at equilibrium in buffer solutions with pH 7.4 and 1.2, simulating physiological conditions. The compression modulus of the conventional and DC hydrogels was measured between 5 and 10% compression, in the linear area of the stress–strain curves (inset of Figure 9). It can be observed that the values of the elastic modulus increased from 2.78 kPa for conventional hydrogel (PNH_0.6_) to 6.54 kPa for DC hydrogel (DC_0.6_–P5). The Young modulus changed directly proportional to the amount of cross-linker, due to the considerable number of bonds between macromolecules in the polymeric network and, consequently, the mobility of macromolecules diminished. However, the elastic modulus values of DC hydrogels were still low, unlike other double cross-linked hydrogels [47], and, thus, highly elastic. Nevertheless, the highest stress value was also observed for the DC_0.6_–P5 with 21.32 kPa in ABS, which was significantly higher, compared with PNH_0.6_ (4.5 kPa) (Figure 9). As a result, the Young’s modulus and maximum stress of the DC hydrogels might provide insight into designing a suitable scaffold for tissue engineering, or other biomedical applications, that require soft and flexible, as well as strong materials [9,12].

### 2.4. In Vitro Release Studies

**Figure 10:**
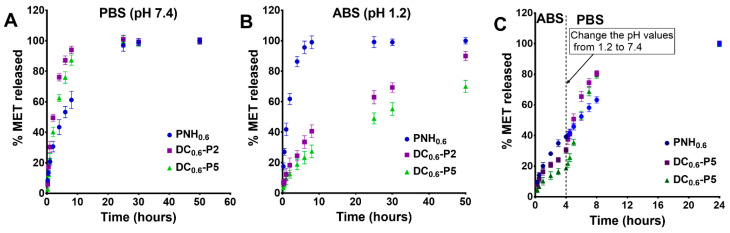
Release kinetics of Met from DC_0.6_ hydrogels in PBS (pH 7.4) (**A**) and ABS (1.2) (**B**), at 37 °C. Met release profile in ABS (pH 1.2) for 4 h and then transferred in PBS (pH 7.4) for 24 h (**C**). For comparison, the release profile of Met from PNH_0.6_ is also depicted (**A**–**C**).

Usually, the delivery of drugs can be controlled by the swelling of the polymeric matrix and diffusion of the entrapped drug [48]. Met was chosen as a drug model to investigate the drug loading/release characteristics of DC hydrogels in comparison with the conventional ones. Met is a cationic molecule with a pKa value of 9.2 [49], and P(MVE/MA) is an anionic macromolecule with pKa values of 3.47 and 6.47 [36]; therefore, at physiological pH (PBS at pH 7.4) both are completely ionized. Drug loading takes place by physical retention of the drug in the polymeric network and mainly by electrostatic interactions between the anionic carboxylic groups of the DC hydrogel and the cationic amino groups of metoclopramide. Therefore, the higher the number of carboxylic groups (exchange capacity) in the hydrogel the more the drug is loaded. As follows, the drug loading capacity increased from 10 mg Met/g for the un-charged conventional hydrogel (PNH_0.6_) to 18 mg Met/g for DC_0.6_–P2 with a lower exchange capacity (1.02 meq/g) and 22 mg Met/g for DC_0.6_–P5 with a higher exchange capacity (1.37 meq/g). On the other side, the covalent cross-linking supposes the involvement of carboxylic groups in the formation of ester bonds and, as a result, reduces their number. Therefore, with the increase in the degree of cross-linking, the capacity to retain the drug through electrostatic interactions should decrease. On the other hand, a more cross-linked network should physically retain more drug. Increasing the degree of cross-linking had two opposite effects that could not be dissociated. The release kinetics of Met from DC were investigated in PBS (pH 7.4) and ABS (pH 1.2), at 37 °C. As can be observed from Figure 10, Met was released faster from the DC, than from the PNH, hydrogel, due to the higher degree of swelling in PBS (ionization of carboxylic groups). At pH = 1.2, the DC hydrogels were fully protonated and the network collapsed. Hydrogels display a delayed drug release, compared to rapid release from the conventional ones. It should be emphasized that Met is much more soluble in pH = 1.2 (hydrochloride form) than in pH = 7.4, so, as a result, the contribution of the polymer network in delaying the release of the drug in the gastric fluid is even more significant. In conclusion, the DC hydrogels exhibited a delayed drug release pattern in acidic media and a faster release in alkaline media. Thus, DC hydrogels, loaded with metoclopramide, could be orally administered. After administration, these polymeric structures first reach the stomach fluid, where the pH is low (1.0–3.0). Here, the residence time is between 2 and 4 h. Then, the gastric chyme (partially digested food) formed in the stomach passes into the small intestine for nutrient absorption (residence time of more than 24 h). To illustrate this theory better, the release kinetics was investigated for 4 h at pH = 1.2 and then continued for another 24 h in PBS (Figure 10). It was obvious that the amount of Met released after 4 h in pH = 1.2 was very small; changing the pH of the buffer from 1.2 to 7.4, the release rate increased significantly.

In order to determine the main release mechanism of Met from hydrogels, the Korsmeyer–Peppas, Higuchi and Zero-order models were applied. The obtained data are listed in Table 3.

The values of the correlation coefficient R^2^ indicate the kinetic model that is appropriate for the release of the drug. A regression coefficient value, R^2^, close to 1 indicates the best model fitting the release mechanism. As shown in Table 3, irrespective of the pH values, the best kinetic model was the Higuchi equation, as the plots showed high linearity (R^2^ values in the range 0.991–0.9998), mainly suggesting a diffusion process. These results were consistent with the data obtained by the Korsmeyer–Peppas model analysis for all hydrogels. According to this model, the drug is transported via Fickian diffusion if the value of the parameter n is below, or equal to, 0.5, while an n value between 0.5 and 0.9 indicates anomalous transport [50]. In an acidic medium, fitting the data to the Higuchi equation, together with n values slightly above 0.5, might suggest a diffusion-controlled drug release, both for conventional and double cross-linked hydrogels. In PBS, *n* values for double cross-linked hydrogels were 0.76 (DC_0.6_–P2) and 0.79 (DC_0.6_–P5), suggesting a coupling of diffusion and anomalous diffusion, which might indicate that Met release was controlled by more than one process (Fickian diffusion and polymer chain relaxation). The *K*_KP_ constant decreased with increase in cross-linking degree, both in acid and basic release mediums.

## 3. Conclusions

Thermo-/pH-responsive double cross-linked hydrogels were prepared in three steps. First, poly(PNIPAAm-co-HEAAm)–based hydrogels (PNHs) were obtained by the free-radical polymerization of NIPAAm and HEAAm in the presence of different amounts of BisAAm, as a cross-linker. In the second step, a semi-interpenetrated hydrogel was obtained, by entrapping linear P(MVE/MA) within the 3D-network of the PNH hydrogels. The last step consisted of double cross-linking of PNH with P(MVE/MA) through a thermal method, without using any solvent. The morphology, swelling degree, and release kinetics of Met in simulated physiological conditions were studied. SEM observations of lyophilized hydrogels showed the presence of filled pores. It must be noted that in PBS at pH = 7.4 the VPTT value increased from 33 °C, for un-charged conventional hydrogels, to 50 °C, for DC hydrogels with the carboxylic groups in the ionized state. However, surprisingly, when the carboxylic groups of the DC hydrogels interacted electrostatically with Met, the VPTT value decreased to 37 °C, a temperature close to that of the human body. This behavior represents a great advantage for the hydrogels, since they are usually designed as biomaterials with potential applications in tissue engineering or drug delivery. The DC hydrogels proved to have higher swelling capacities in a basic medium. On the contrary, in the acidic medium, the DC hydrogels had lower swelling capacities compared to conventional ones. Moreover, the DC hydrogels showed a rapid and controllable deswelling process by complexation with Met. Contrary to the fast release rate of Met from conventional hydrogels in gastric fluid, the DC hydrogels displayed a delayed release pattern in acidic medium and a faster release in the alkaline medium. Thus, DC hydrogels loaded with metoclopramide could be administered orally. Moreover, the initial concentration of P(MVE/MA) in hydrogel could be adjusted to tailor for the desired properties and to obtain scaffolds for different tissue engineering applications.

## 4. Materials and Methods

### 4.1. Materials

*N*-isopropylacrylamide (NIPAAm) and hydroxyethylacrylamide (HEAAm) were bought from Sigma-Aldrich Chemie Gmbh, Darmstadt, Germany. Potassium persulfate (KPS), *N*,*N*′-methylenebisacrylamide (BisAAm) and *N*,*N*,*N*′,*N*′-tetramethylethylenediamine (TEMED) were acquired from Fluka, Buchs, Switzerland. Poly(methyl vinyl ether-co-maleic anhydride) (P(MVE/MA)) was purchased from Aldrich, Germany). Metoclopramide (Met) was purchased from Sigma-Aldrich Chemical Co. (St. Louis, MO, USA). The phosphate buffer solutions (PBS) at pH 7.4 (50 mM NaH_2_PO_4_ + 40 mM NaOH) and acidic buffer solutions (ABS) at pH 1.2 (50 mM KCl + 64 mM HCl) were prepared in our laboratory.

### 4.2. Methods

#### 4.2.1. Synthesis of Conventional and Double Cross-Linked Hydrogels

The synthesis of conventional hydrogels (PNHs) was carried out as follows: 0.565 g of NIPAAm (5 mmol), 0.115 g of HEAAm (1 mmol) and varied amounts of BisAAm from 0.4 to 0.8% mol vs. monomers were dissolved in 5 mL distilled water. Dry nitrogen was bubbled through the solution for 30 min before polymerization. Next, the initiator (0.03 g, 0.111 mmol of KPS; 1.85% mol KPS vs. monomers) was added and the solution kept for 5 min under nitrogen atmosphere to generate free radicals. Finally, 100 µL of TEMED were added to accelerate the polymerization, then the solution was poured fast into a 5 mL syringe (15 mm diameter). The reaction mixture was left for 24 h for a complete polymerization of the PNH. The obtained samples were extracted from the syringe and cut at a 10 mm width.

In order to obtain double cross-linked hydrogels, the PNH were immersed in a P(MVE/MA) solution of different concentrations (2 and 5%, *w*/*v*) for 48 h at ambient temperature. Subsequently, the hydrogel samples were removed from the solution, wiped with a filter paper, and then rapidly frozen in liquid nitrogen and lyophilized (−57 °C, 5.5 × 10^−4^ mbar) for 48 h. The chemical cross-linking took place in solid state by thermal treatment at 80 °C in a vacuum oven.

#### 4.2.2. Scanning Electron Microscopy (SEM) Analysis

The SEM analyses were achieved on lyophilized samples with an Environmental Scanning Electron Microscope (ESEM, Quanta 200, in Low Vacuum, at 20 kV).

#### 4.2.3. Attenuated Total Reflectance-Fourier Transform Infrared (ATR-FTIR) Spectroscopy

The IR spectra of lyophilized samples were registered using a FT–IR Vertex 70 spectrophotometer (Bruker, Wien, Austria, with KRS-5 ATR accessory). The frequency ranged between 4000 and 600 cm^−1^ and the resolution was 4.0 cm^−1^. To obtain the spectra, the average of 128 scans was used and the obtained data were processed with the OPUS 6.5 software (Bruker Optics, Wien, Austria).

#### 4.2.4. Conductometric Titration

The carboxyl groups in DC hydrogels were quantitatively determined by conductometric titration. A conductivity meter CMD 210 (Radiometer, Copenhagen, Denmark) provided with a CDC 865 cell was used. Previously, the DC hydrogels were weighed and placed in an excess of 0.1 M NaOH aqueous solution. After the ion exchange reached equilibrium, the samples were titrated with 0.1 M HCl solution. The exchange capacity was determined from the graphical representation of the conductivity variation with the HCl volume added. The second cross-linking degree of DC hydrogel was also determined from the exchange capacity.

#### 4.2.5. Porosity Measurement

Porosity of both conventional and DC hydrogels was determined using the solvent replacement method. Thus, cylinder-like samples of dried hydrogels were weighed and submerged in absolute ethanol (purity > 99.9%) for 100 h. After that, the samples were weighed. To remove the excess of ethanol on the surface, the samples were wiped with a filter paper. In addition, the dimensions of the hydrogel discs were evaluated [51,52]. The porosity was calculated according to the following equation:

(1) $$P\%=\frac{W_{1}-W_{2}}{\rho V}$$

where $W_{1}$ and $W_{2}$ are the weight (g) of the samples before and after soaking in absolute ethanol, respectively; ρ is the density of absolute ethanol (g/cm^3^) and V is the volume of the DC samples (cm^3^).

#### 4.2.6. Mechanical Tests

The compressive experiments were realized at room temperature using a Texture Analyzer (Brookfield Texture PRO CT3^®^, Middleboro, MA, USA). To compress the samples into disks with radius and thickness of around 8 mm, a strain rate of 50% min^−1^ was used. Testing was carried out at a speed of 30 mm/min. The Young’s modulus of hydrogel samples was obtained from the slope of the stress–strain plot.

#### 4.2.7. Swelling Studies

The swelling ratio (SR) of both PNH and DC hydrogel samples was studied over a 22–60 °C range with 3–5 °C heating rate, using a thermostatic water bath. Previously, the dry samples were weighed and then left in buffer solution to equilibrate for 12 h at each predefined temperature. To remove the excessive solution, the samples were wiped with filter paper before each weighing. The swelling ratio was calculated according to the following equation:

(2) $$SR=\frac{W_{s}-W_{d}}{W_{d}}$$

where SR is the swelling ratio, $W_{s}$ is the mass of swollen sample at each temperature and $W_{d}$ is the mass of dry sample. The dry mass of samples ($W_{d}$) was achieved after drying at room temperature and subsequent vacuum drying for 24 h.

The VPTT of hydrogels was determined as the inflexion point in the curve swelling factor-temperature by means of Boltzmann fitting of the experimental data.

##### Swelling/Deswelling Kinetics

The swelling/deswelling kinetics in simulated physiological conditions were studied by the gravimetric method. For swelling measurements, the dry hydrogel sample was weighed and then immersed in an excess of buffer solution at room temperature (21 °C). For deswelling experiments, the samples were first left in buffer solutions at room temperature until the equilibrium swelling degree was reached. Subsequently, the samples were rapidly placed in hot solutions at 50 °C. For both swelling and deswelling analysis, at predefined time intervals, the samples were taken out from the solution, wiped with a filter paper and then weighed.

The volume change of DC hydrogels with pH variation was evaluated at 37 °C by shifting PBS (pH 7.4) with ABS (pH 1.2) as the swelling media. Previously, the samples were weighed and placed in PBS for 2 h (being weighed at each predefined time point). Subsequently, the samples were moved to the ABS solution and weighed following the same operation as in PBS. The buffer solution was changed every 2 h.

#### 4.2.8. Drug Loading and Release Studies

For drug loading, each dried sample (~0.180 g) was placed in 5 mL of Met aqueous solution (1 mg/mL) for three days at room temperature. Then, the loaded hydrogels were washed with distilled water and left to dry at room temperature, followed by vacuum oven drying. The amount of loaded Met was estimated from the amount of drug in the washing water (UV determination) according to the following equation:

(3) $$Met_{loaded}=Met_{i}-Met_{r}$$

where $Met_{i}$ and $Met_{r}$ are the amount of *Met* in the initial and final solution, respectively.

In vitro release studies were performed in PBS (pH 7.4) and ABS (pH 1.2) at 37 °C. A quantity of 0.180 g of sample containing 3 mg of *Met* was placed in a buffer solution. In order to determine the amount of drug released, 3 mL from the release medium were extracted, at predefined time points, and analyzed spectrophotometrically at 272 nm. 3 mL of fresh buffer solution was added after each extraction to keep the volume of the release medium constant.

The data from the release kinetics of *Met* from conventional and DC hydrogels were fitted to the following mathematical model:

(4) $$\mathrm{Korsmeyer}–\mathrm{Peppas}:\;\frac{M_{t}}{M_{\infty }}=K_{KP}t^{n}$$

(5) $$\mathrm{Higuchi}:\;\frac{M_{t}}{M_{\infty }}=K_{H}t^{0.5}$$

(6) $$\mathrm{Zero}-\mathrm{Order}:\;\frac{M_{t}}{M_{\infty }}=K_{ZO}t$$

where:

-$M_{t}/M_{\infty }$ is the fraction of drug released at time t,

-$K_{KP}$, $K_{H}$, $K_{KO}$ are the Korsmeyer-Peppas, Higuchi, and Zero-Order constant, respectively,

-n is the release exponent indicative for the drug release mechanism. The drug release mechanism was the following: Fickian (Case I) for a value around 0.5 and non-Fickian (anomalous) diffusion for a value between 0.5 and 0.9 and in Case II transport for a value around 0.9 [50].

Experimental results were expressed as mean ± SD (n = 3).

## Figures and Tables

**Table 1:** Composition of the initial reaction mixture and of DC hydrogels.

Sample	Comonomer Composition in the Feed ^a^			
	10^−3^ M		(% moles) ^b^	
	NIPAAm	HEAAm	BisAAm	
PNH_0.4_	5	1	0.4	
PNH_0.6_	5	1	0.6	
PNH_0.8_	5	1	0.8	
	**Conventional hydrogels used to obtain DC** **hydrogels**		**P(MVE/MA)**	
			**in the feed solution** **(%, *g*/*v*)**	**in the final hydrogel** **(% *g*/*g*)**
DC_0.4_–P2	PNH_0.4_		2	25.88 ± 2.4
DC_0.4_–P5	PNH_0.4_		5	44.57 ± 1.8
DC_0.6_–P2	PNH_0.6_		2	25.55 ± 2.2
DC_0.6_–P5	PNH_0.6_		5	40.67 ± 1.9
DC_0.8_–P2	PNH_0.8_		2	22.90 ± 2.1
DC_0.8_–P5	PNH_0.8_		5	34.58 ± 1.5

^a^ Monomers were polymerized by the radical initiator KPS (2% to monomers) in water (5 mL). ^b^ vs. total mols of comonomers.

**Table 2:** The main characteristics of the most relevant DC hydrogels in comparison with conventional ones.

Samples	Porosity (%)	Second Cross-Linking Degree (%) *	Exchange Capacity (meq/g)
PNH_0.6_	95.44	-	-
DC_0.6_–P2	84.27	10.52	1.02
DC_0.6_–P5	74.52	25.35	1.37

Data were the results of three independent experiments. * The cross-linking degree was determined by dividing the difference between the theoretical and effective exchange capacity to the theoretical exchange capacity.

**Table 3:** Release parameters corresponding to Met-loading hydrogels.

Samples		Korsmeyer-Peppas			Higuchi		Zero Order Model	
		*K*_KP_ × 10^2^ (Hours ^−n^)	*n*	R^2^	*K*_H_ × 10^2^ (Hours ^−1/2^)	R^2^	*K*_0_ × 10^2^ (Hours^−1^)	R^2^
PNH_0.6_	ABS	41.06	0.59	0.999	0.46	0.998	0.042	0.8747
	PBS	19.64	0.56	0.996	0.22	0.992	0.066	0.9578
DC_0.6_–P2	ABS	11.84	0.59	0.994	0.15	0.991	0.039	0.9862
	PBS	29.60	0.76	0.998	0.46	0.999	0.110	0.9063
DC_0.6_–P5	ABS	8.51	0.55	0.996	0.10	0.999	0.025	0.967
	PBS	21.90	0.79	0.994	0.46	0.996	0.100	0.9449

## Data Availability

The data presented in this study are available on request from the corresponding author.
